# Real-world cost-effectiveness of rivaroxaban compared with vitamin K antagonists in the context of stroke prevention in atrial fibrillation in France

**DOI:** 10.1371/journal.pone.0225301

**Published:** 2020-01-24

**Authors:** Kevin Bowrin, Jean-Baptiste Briere, Laurent Fauchier, Craig Coleman, Aurélie Millier, Mondher Toumi, Emilie Clay, Pierre Levy

**Affiliations:** 1 Bayer Plc, Reading, England, United Kingdom; 2 Bayer AG, Berlin, Germany; 3 Cardiologie, Centre Hospitalier Universitaire Trousseau et Université François Rabelais, Tours, France; 4 University of Connecticut, School of Pharmacy, Storrs, Connecticut, United States of America; 5 Creativ-Ceutical, Paris, France; 6 Aix-Marseille University, Marseille, France; 7 Université Paris-Dauphine, PSL Research University, LEDa-LEGOS, Paris, France; Leiden University Medical Center, NETHERLANDS

## Abstract

**Objective:**

The objective was to assess the real-world cost-effectiveness of rivaroxaban, versus vitamin K antagonists (VKAs), for stroke prevention in patients with atrial fibrillation (AF) from a French national health insurance perspective.

**Methods:**

A Markov model was developed with a lifetime horizon and cycle length of 3 months. All inputs were drawn from real-world evidence (RWE) studies: data on baseline patient characteristics at model entry were obtained from a French RWE study, clinical event rates as well as persistence rates for the VKA treatment arm were estimated from a variety of RWE studies, and a meta-analysis provided comparative effectiveness for rivaroxaban compared to VKA. Model outcomes included costs (drug costs, clinical event costs, and VKA monitoring costs), quality-adjusted life-years (QALY) and life-years (LY) gained, incremental cost per QALY, and incremental cost per LY. Sensitivity analyses were performed to test the robustness of the model and to better understand the results drivers.

**Results:**

In the base-case analysis, the incremental total cost was €714 and the total incremental QALYs and LYs were 0.12 and 0.16, respectively. The resulting incremental cost/QALY and incremental cost/LY were €6,006 and €4,586, respectively. The results were more sensitive to the inclusion of treatment-specific utility decrements and clinical event rates.

**Conclusions:**

Although there is no official willingness-to-pay threshold in France, these results suggest that rivaroxaban is likely to be cost-effective compared to VKA in French patients with AF from a national insurance perspective.

## Introduction/Background

Atrial fibrillation (AF) is a cardiac arrhythmia with structural and/or electrophysiological abnormalities that induce remodelling in the atria; it is the most common cardiac arrhythmia [1–3]. Worldwide, an estimated 3% of adults aged 20 years or older suffer from AF, approximately 20.9 million men and 12.6 million women [2]. Due to the aging of the population, the worldwide prevalence is predicted to at least double in many countries during the next several decades [4, 5].

AF is associated with substantial morbidity and mortality [2]. Compared to otherwise healthy individuals, men and women with AF are at a 1.5-fold and 2-fold increased risk for all-cause mortality, respectively [2], and the risk for strokes increased by 2-to-7-fold [4]. Strokes are associated with significant financial burden [6]; in 2015, the total costs of stroke care in the European Union (EU) was estimated to be €45 billion euros [7]. It is expected that between 2015 and 2035, there will be a 34% increase in total number of stroke events in the European Union [7].

Oral anticoagulants including vitamin K antagonists (VKAs) or non-VKA oral anticoagulants (NOACs) such as rivaroxaban, dabigatran, apixaban, and edoxaban, have been established as a cornerstone of management in patients with AF and to reduce stroke incidence and mortality [2] in randomized clinical trials (RCTs) [2].

Many uncertainties remain regarding the relevance of the results of RCTs in a real-world setting. Real-world evidence (RWE) may provide additional information to decision-makers [8]. Indeed, RWE sample size is not limited as it is the case of RCTs. RCTs have to respect inclusion/exclusion criteria regarding population selection. Also, RWE can offer long-term outcomes while the timeframe of RCT is usually shorter with only a few outcomes [9].

A meta-analysis comparing NOACs with VKAs and reporting effectiveness, safety, and persistence using RWE has recently been published [10]. It confirmed the findings of rivaroxaban pivotal RCT [11] and concluded that rivaroxaban is a suitable alternative to VKAs in routine clinical practice. Health Technology Assessment (HTA) agencies are frequently requesting manufacturers to prove the benefits of their health technology in the real-world, not only in terms of clinical RWE but also in terms of RWE cost-effectiveness. Indeed, RWE is of interest since it reflects more closely what happens in a real-world setting. While many initial coverage and reimbursement decisions are based on cost-effectiveness models using RCT efficacy and safety data, the use of RWE can provide more realistic estimates of cost-effectiveness based on how the drug is being used in clinical practice, its effectiveness, safety, and associated costs. The availability of a RWE meta-analysis provides a good opportunity to evaluate the RWE cost-effectiveness of rivaroxaban compared to VKAs for the prevention of stroke in patients with AF.

In France, NOACs are acknowledged as an important component of the national stroke plan, but there is an increasing scrutiny regarding the cost of these therapies; therefore, a French national healthcare insurance (NHI) perspective was considered relevant to demonstrate the real-world value of these therapies.

## Methods

### Model approach

**Fig 1:**
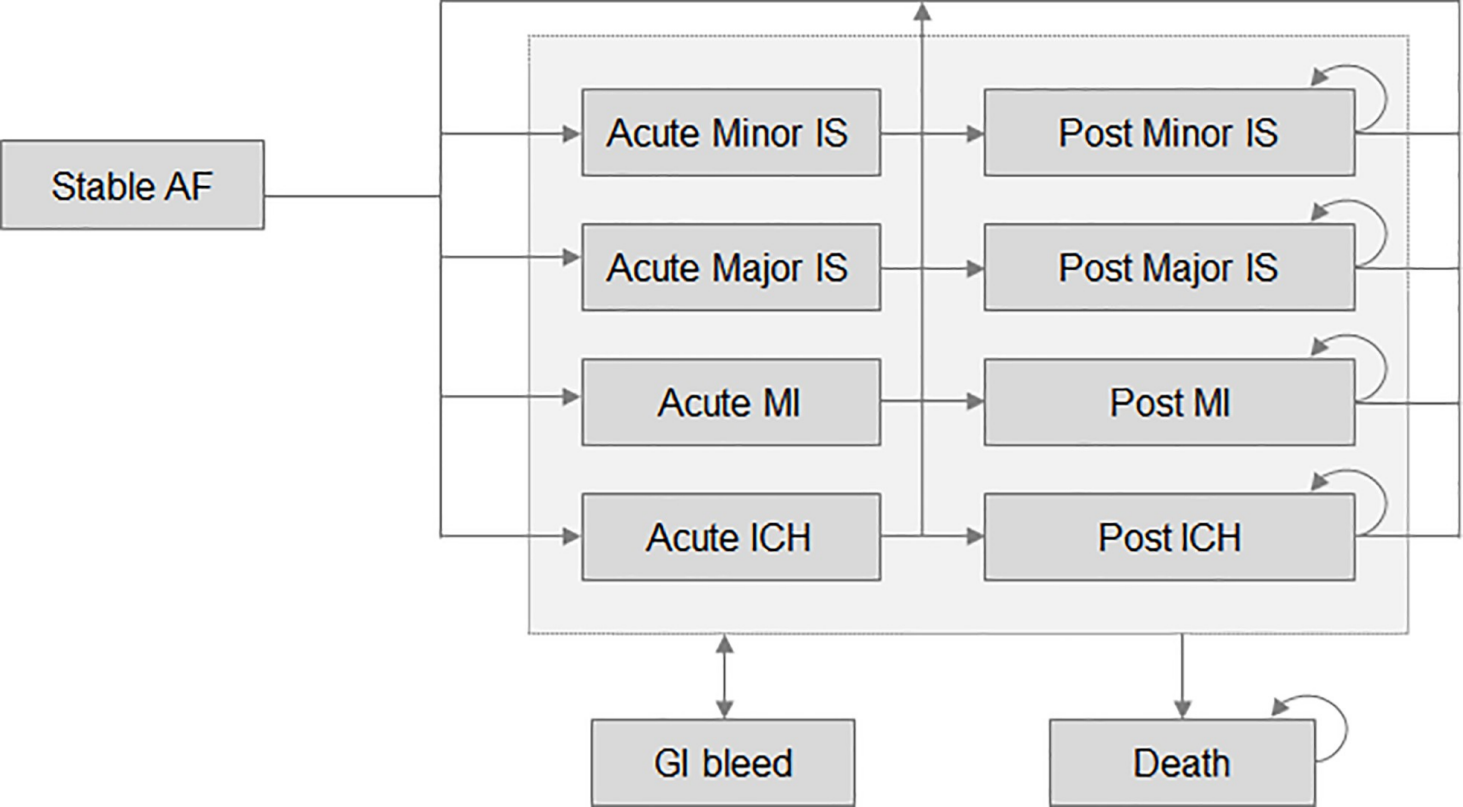
Model diagram. Abbreviations: AF, atrial fibrillation; GI, gastrointestinal; ICH, intracranial haemorrhage; IS, ischaemic stroke; MI, myocardial infarction.

An already published Markov cost-effectiveness model [12] was adapted to assess the incremental costs and health outcomes of rivaroxaban compared to VKA in patients with AF in real-world settings (Fig 1). Patients enter the model initiating a first-line treatment with either rivaroxaban or VKA, and could progress between health states according to transition probabilities. Health states included stable AF, acute and post major ischaemic stroke (IS), acute and post minor IS, acute and post myocardial infarction (MI), acute and post intracranial haemorrhage (ICH), gastrointestinal (GI) bleed, and death. Patients were always at a risk of an event; however, the model assumed that only one event could occur per cycle. Additionally, long term consequences of an event were considered until death or until the occurrence of a subsequent event with long-term consequences. All patients could either be on-treatment (rivaroxaban, initial VKA or other VKA after a switch) or off-treatment (once all treatments are discontinued). Indeed, the model allowed all patients to discontinue their initial treatment, to switch (from rivaroxaban to VKA, from VKA to other VKA), or to stop treatment (from rivaroxaban or VKA to no treatment). Patients who had switched or discontinued treatment could still experience any clinical event.

The model outcomes included the number of IS, the number of MI, and the number of bleeds, as well as the total quality-adjusted life-years (QALY), the total life-years (LY) gained, the total costs and the incremental cost per QALY or per LY gained. All model inputs as presented in Table 1 were drawn from RWE studies.

**Table 1:** Model inputs: Three-month probabilities of events and discontinuation, mortality, utility and costs.

	Value	Range used in DSA	Distribution used in PSA	Source
**Three-month probabilities (VKA arm)**				
Minor IS	0.151%	[0.146%; 0.155%]	Beta (4,539; 3,005,582)	Weighted average of event rates identified in Coleman et al. 2019 [10] + Hylek et al. 2003 [13]
Major IS	0.217%	[0.211%; 0.223%]	Beta (4,536; 2,085,855)	
MI	0.193%	[0.181%; 0.205%]	Beta (1,037; 536,223)	
GI bleed	0.406%	[0.395%; 0.417%]	Beta (5,469; 1,341,752)	
ICH	0.199%	[0.190%; 0.208%]	Beta (1,778; 891,500)	
Discontinuation				
0–3 months	29.10%	[28.63%; 29.57%]	Beta (10,283; 25,053)	Coleman et al. 2016 [14]
3–6 months	17.07%	[16.67%; 17.46%]	Beta (6,031; 29,305)	
6–12 months	15.48%	[15.11%; 15.86%]	Beta (5,472; 29,864)	
12+ months	10.88%	[10.55%; 11.20%]	Beta (3,843; 31,493)	
Proportion of switch among discontinued patients	16.10%	[13.06%; 19.14%]	Beta (90; 469)	Collings et al. 2018 [15]
**Three-month probabilities (rivaroxaban arm)**				
Minor IS	0.125% (HR: 0.83)	[0.113%; 0.140%] (HR: [0.75;0.93])	Beta (343; 267,304)	HRs from Coleman et al. 2019 [10] applied to tree-month probabilities of VKA arm
Major IS	0.180% (HR: 0.83)	[0.163%; 0.202%] (HR: [0.75;0.93])	Beta (343; 185,544)	
MI	0.185% (HR: 0.96)	[0.154%; 0.220%] (HR: [0.80;1.14])	Beta (135; 73,678)	
GI bleed	0.495% (HR: 1.22)	[0.455%; 0.540%] (HR: [1.12;1.33])	Beta (472; 94,947)	
ICH	0.137% (HR: 0.69)	[0.104%; 0.179%] (HR: [0.52;0.90])	Beta (69; 51,161)	
Discontinuation				
0–3 months	18.04% (HR: 0.62)	[17.46%; 18.92%] (HR: [0.60;0.65])	Beta (1462; 6,268)	
3–6 months	10.58% (HR: 0.62)	[10.24%; 11.09%] (HR: [0.60;0.65])	Beta (1603; 12,849)	
6–12 months	9.60% (HR: 0.62)	[9.29%; 10.06%] (HR: [0.60;0.65])	Beta (1622; 14,492)	
12+ months	6.74% (HR: 0.62)	[6.53%; 7.07%] (HR: [0.60;0.65])	Beta (1676; 22,032)	
Proportion of switch among discontinued patients	12.00%	[7.64%; 16.36%]	Beta (25; 187)	Collings et al. 2018 [15]
**Three-month probabilities (no treatment arm)**				
Minor IS	0.437% (HR: 2.91)	[0.274%; 0.698%] (HR: [1.82; 4.66])	Beta (27; 6,247)	Unpublished HRs applied to three-month probabilities of VKA arm
Major IS	0.629% (HR: 2.91)	[0.394%; 1.005%] (HR: [1.82; 4.66])	Beta (27; 4,324)	
MI	0.453% (HR: 2.35)	[0.147%; 1.610%] (HR: [0.76; 8.40])	Beta (8; 1,837)	
GI bleed	0.094% (HR: 0.23)	[0.024%; 0.296%] (HR: [0.06; 0.73])	Beta (7; 7,507)	
ICH	0.036% (HR: 0.18)	[0.006%; 0.156%] (HR: [0.03; 0.77])	Beta (6; 15,416)	
**In-hospitalisation mortality rates per clinical event in model**				
Minor IS	0.00%	-	-	Assumption
Post minor IS	0.00%	-	-	Assumption
Major IS	13.13%	[12.66%; 13.60%]	Beta (2,614; 17,294)	Fauchier et al. 2015 [16]
Post major IS	8.12%	[7.35%; 8.92%]	Beta (390; 4,410)	Lip et al. 2015 [17]
MI	8.28%	[7.05%; 9.51%]	Beta (159; 1,760)	Blin et al.2016 [18]
Post-MI	8.24%	[7.17%; 9.34%]	Beta (211; 2,347)	Lip et al.2015 [17]
ICH	18.77%	[16.96%; 20.58%]	Beta (335; 1,449)	Fauchier et al. 2015 [16]
Post -ICH	14.11%	[11.85%; 16.57%]	Beta (128; 781)	Lip et al.2015 [17]
GI bleed	5.38%	[3.90%; 6.85%]	Beta (48; 844)	Cotte et al. 2014 [19]
**Utility values**				
Stable	0.73	[0.71; 0.75]	Beta (1598;600)	Kongnakorn 2015 et al. [20]
Minor IS	0.73	[0.56; 0.68]	Beta (163; 100)	Luengo-Fernandez et al. 2013 [21]
Major IS	0.41	[0.48; 0.60]	Beta (149; 127)	
Post minor IS	0.73	[0.45; 0.74]	Beta (24;17)	
Post major IS	0.56	[0.20; 0.34]	Beta (45;121)	
MI	0.66	[0.55; 0.67]	Beta (162; 103)	Pockett et al. 2014 [22]
Post-MI	0.73	[0.46; 0.77]	Beta (23; 15)	
ICH	0.56	[0.61; 0.69]	Beta (367; 196)	Luengo-Fernandez et al. 2013 [21]
Post-ICH	0.67	[0.47; 0.51]	Beta (999; 1044)	
GI bleed	0.70	[0.69; 0.72]	Beta (3287;1395)	Kongnakorn 2015 et al. [20]
**Costs (€)**				
**Drug costs**				
Acquisition VKA (daily)	0.13	-	-	AMELI [23]
Acquisition rivaroxaban (daily)	2.02	-	-	AMELI [24]
Monitoring VKA (cycle)	80.86	[64.27; 107.12]	Gamma (55; 1.4)	Data on file
Monitoring rivaroxaban (cycle)	15.57	[11.67; 19.46]	Gamma (61; 0.3)	Data on file
**IS**				
Acute treatment (minor)	3,975	[2,783; 5,158]	Gamma (43; 93)	Lanitis et al. 2014 [25], based on Cotte et al. 2014 [19]
Acute treatment (major)	12,574	[8,802; 16,346]	Gamma (43; 295)	
Monthly follow-up (minor)	595	[417; 774]	Gamma (43; 14)	
Monthly follow-up (major)	2,382	[1,667; 3,096]	Gamma (43; 56)	
Rehabilitation	10,112	[7,584; 12,640]	Gamma (61; 165)	Cotté et al. 2016 [26] (using stroke/TIA/SE as a proxy)
**MI**				
Acute Treatment (one event per cycle)	4,289	[3,002; 5,576]	Gamma (43; 100)	Lanitis et al. 2014 [25], based on Cotte et al. 2014 [19]
Monthly Follow-up	1,045	[732; 1,359]	Gamma (43; 25)	
**Bleeds**				
Acute treatment GI bleed (non-ICH)	2,952	[2,066; 3,838]	Gamma (43; 69)	Lanitis et al. 2014 [25], based on Cotte et al. 2014 [19]
Acute treatment—ICH	10,347	[7,243; 13,452]	Gamma (43; 242)	
Monthly follow-up	2,382	[1,667; 3,096]	Gamma (43; 56)	Assumption (set as equivalent to cost of major IS follow-up)
Rehabilitation	6,300	[2,066; 3,838]	Gamma (62; 102)	Cotté et al. 2016 [26]
**Resource use for rehabilitation**				
% of rehabilitation for minor IS	34.8%	[34.1%; 35.5%]	Beta (6,928; 12,980)	Cotté et al. 2016 [26]
% of rehabilitation for major IS	34.8%	[34.1%; 35.5%]	Beta (6,928; 12,980)	
% of rehabilitation for GI bleed	14.2%	[13.4%; 15.0%]	Beta (1,063; 6,421)	
% of rehabilitation for ICH	32.9%	[31.5%; 34.3%]	Beta (1,391; 2,836)	

Abbreviation: AMELI, official French Health Insurance website; DSA, deterministic sensitivity analysis; GI, gastro-intestinal; HR, hazard ratio; ICH, intracranial haemorrhage; IS, ischaemic stroke; MI, myocardial infarction; PSA, probabilistic sensitivity analysis; VKA, vitamin K antagonist.

### Cycle length and time horizon

The model considered a lifetime time horizon (30 years) in order to fully capture the expected long-term costs and health effect consequences of AF in accordance with French guidelines [27]. The model cycle length was set to 3 months which was assumed sufficient to enable the capture of short-term events and their acute impact on costs and clinical outcomes.

### Study perspective

This analysis was conducted from the French NHI perspective. The model was discounted at a rate of 4% for costs and benefits as recommended by pharmacoeconomic guidelines in France [27].

### Patients’ characteristics

In order to ensure generalisability to the French AF population, the model was populated with clinical characteristics drawn from RWE studies, representative of the French AF population for which NOACs are indicated [28]. Patients entered the model at a mean age of 70 years, 15% had an intermediate CHA_2_DS_2_-VASc score (= 1) and 85% had a high CHA_2_DS_2_-VASc score (≥2).

### Clinical data implementation

RWE was used to inform clinical event rates for VKA, while rates for rivaroxaban were estimated by applying relevant RWE hazard ratios (HRs) to the VKA transition probabilities retrieved from the RWE meta-analysis on prevalent and incident patients [10]. Clinical event rates for patients using “other VKA” were assumed to be the same as for the initial VKA.

All studies included in the RWE meta-analysis provided annual rates for the VKA arm of the model. All retrieved annual rates were pooled using weighted means by sample size as weights, and were then converted to 3-month probabilities before the implementation in the model. As the model distinguishes between major and minor IS, it was necessary to consider the proportions of 59% of IS as major and 41% as minor [13]. Additionally, VKA risk for IS was adjusted by age according to the risk score calculator derived from the Framingham Heart Study [29]. The relative risk of IS for patients aged 70–74 years was set at 1.0; IS risk for all other age groups was calculated in relation to this subgroup (Table 2). Finally, the stroke risk was increased by 1.53 after a stroke in line with the results obtained in the XANTUS observational study [30].

**Table 2:** Relative risk for ischaemic strokes by age group [29].

Age group	Relative risk
55–59	0.667
60–64	0.760
65–69	0.854
70–74	1.000
75–79	1.146
80–84	1.281
85–89	1.480
90+	1.719

For each treatment, discontinuation probabilities were divided into different time periods (initiation-3 months, 3 months-6 months, 6 months-1 year, and >1 year). For the VKA arm, treatment discontinuation and switch rates were derived from RWE studies [10, 15]. For the rivaroxaban arm, the HR of discontinuation from the RWE meta-analysis and the switch rate from a RWE French study [10, 15] were used.

Death rates based on French life tables, were applied to all modelled states. A number of health states included an additional specific event-related mortality risk, as identified from a literature review on RWE studies in France [16–19], assuming no event-related mortality followed hospital discharge.

### Utility

Utility values were derived from recent European studies [20–22, 31] as no French data were identified. No utility decrements associated with specific treatments were considered in the base case which was explored in a scenario analysis.

### Estimation of costs

Through the third-party payment system, patients with long-term illnesses (LTI) do not pay medical expenses. The LTI coverage was estimated to be 71.6% in these patients [32]; a 100% reimbursement rate including drug international normalized ratio (INR) testing and visit costs was assumed, while a 65% reimbursement rate was applied for non-LTI patients for drugs, 70% for practitioner visits, and 80% for hospital care.

Three relevant cost categories were identified: drug acquisition costs, administration costs (including monitoring and other costs), and clinical event-related costs.

VKA monitoring costs include direct medical costs (INR testing, pharmacy/physician consultations, and travel costs for providers to provide care and/or INR monitoring at the patient’s home) and direct non-medical costs (patient/caregiver travel costs to labs, physician offices, and pharmacy); this additional cost attributable to VKA therapy has been amounted to €81 per cycle [33].

NOACs monitoring costs related to renal and hepatic monitoring include one at-home nurse consultation and additional relevant biology tests, amounted to €16 per cycle. A GP visit was also considered as part of administration costs.

Clinical events unit costs were collected from RWE French studies [25, 26, 34]. Costs were adjusted using the consumer price index in Health sector from the National Institute of Statistics and Economic Studies. All costs have been inflated to €2017 using the consumer price index from the National Institute of Statistics and Economic Studies for adjustment when necessary.

### Sensitivity analyses

A series of univariate deterministic sensitivity analyses (DSA) were run for the base case in order to determine the significant drivers of cost-effectiveness. Otherwise, the confidence interval was used as lower and upper bound of the DSA. When not available, a ±25% variation of the base value was applied for low and high values.

A stochastic component was included in the model to allow multivariate probabilistic sensitivity analyses (PSA) with 2000 iterations. All parameters with second-order uncertainty were included in the PSA. Parameters that did not carry second-order uncertainty were excluded (discount rates, time horizon, unit costs from published reference lists, and patient’s characteristics such as age and co-morbidities). A beta distribution was used for probabilities, utility values, and proportion; whereas, gamma was considered for cost. The variation used in the DSA was used as the confidence interval (CI) to estimate the distribution parameters. The PSA also allowed providing 95% CI to all model health outputs as well as incremental cost-effectiveness ratios (ICERs).

To confirm the validity of the findings, the impact of several assumptions on the results was explored through scenario analyses. A first scenario considered the inclusion of utility decrements related to treatment: an utility decrement of 0.013 was associated with VKA and an utility decrement of 0.002 was associated with rivaroxaban therapy [35]. A second scenario considered HRs from the RWE meta-analysis considering incident patients only. Indeed, bleeding usually occurs in the initial phases of anticoagulant treatment [36], so it could be less common in prevalent than incident patients. Thirdly, alternative probabilities for switches after initial treatment discontinuation were tested (0%, i.e. all patients move to no treatment after their initial treatment, and 100% i.e. all patients have another treatment after their initial treatment before discontinuing to no treatment). Finally, a scenario using the hazard ratios from the ROCKET-AF trial [11] was presented to explore the impact of using RCT data instead of RWE, when it comes to comparative effectiveness.

## Results

### Rivaroxaban vs VKAs

In the base-case analysis, from the NHI perspective, patients treated with rivaroxaban experienced incremental gains in both QALYs (0.12, 95% CI: [0.056; 0.178]) and LYs (0.16, 95% CI: [0.120; 0.189]) compared with VKAs. Treatment with rivaroxaban was associated with fewer MI (0.141, 95% CI: [0.139; 0.144] for rivaroxaban vs 0.148, 95% CI: [0.147; 0.149] for VKA), and a lower rate of IS (0.389, 95% CI: [0.375; 0.405] for rivaroxaban vs 0.414, 95% CI: [0.399; 0.430] for VKA), but was associated with a higher bleeding rate (0.112, 95% CI: [0.107; 0.117] for rivaroxaban vs 0.092, 95% CI: [0.091; 0.093] for VKA). Over a lifetime horizon, costs were higher for rivaroxaban compared with VKA (€15,896, 95% CI: [14,184; 17,821] vs €15,182, 95% CI: [13,415; 17,152]), with incremental costs reaching €714, 95% CI: [271; 1,200]. This is largely due to the higher drug acquisition costs (+€1,604); however, savings were associated with the reduced rates of events (-€745) and drug administration costs (-€146). From the NHI perspective, the base-case analysis resulted in an incremental cost/QALY of €6,006, 95% CI: [2,212; 14,616] and an incremental cost/LY of €4,586, 95% CI: [1,717; 8,286] as summarized in Table 3.

**Table 3:** Model results.

Outcome	Rivaroxaban	VKA	Incremental
**Costs**			
Drug Acquisition costs	€1,696	€91	€1,604
Drug Administration costs	€619	€765	-€146
Event Treatment costs	€13,582	€14,327	-€745
Total costs	€15,896	€15,182	€714
**Health benefits**			
Total QALYs	7.11	7.00	0.12
Total LY	9.91	9.76	0.16
Ischaemic strokes	0.389	0.414	-0.025
Myocardial infarctions	0.141	0.148	-0.007
Bleeds	0.112	0.092	0.020
**Incremental costs-effectiveness ratios**			
Incremental cost/QALY	-	-	€6,006
Incremental cost/LY	-	-	€4,586

Abbreviation: LY, life-year; NHI, National Health Insurance; QALY, quality-adjusted life-year; VKA, vitamin K antagonist.

### Sensitivity analysis

**Fig 2:**
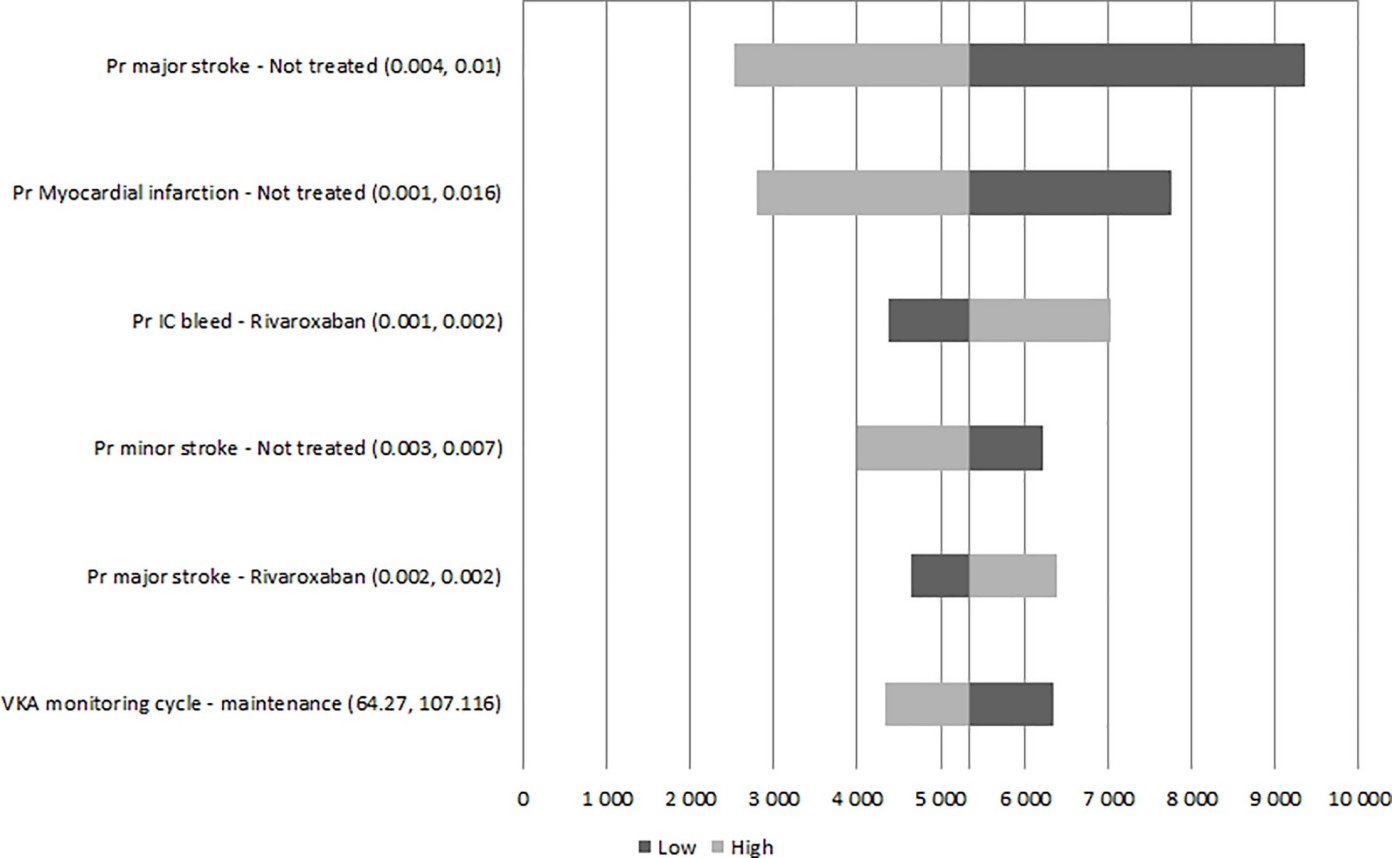
Deterministic sensitivity analysis results (Tornado diagram). Abbreviations: ICER, incremental cost-effectiveness ratio; ICH, intracranial haemorrhage; IS, ischaemic stroke; MI, myocardial infarction; Prob, probability; QALY, quality-adjusted life-year; VKA, vitamin K antagonist.

Univariate sensitivity analyses were performed on all variables (Fig 2). The Tornado graph showed that the results were robust to plausible changes in the inputs parameters with a majority of parameters having a minimal impact on the results. The main drivers identified were the probability of major IS when not treated, the probability of MI when not treated, and the probability of ICH when treated with rivaroxaban.

**Fig 3:**
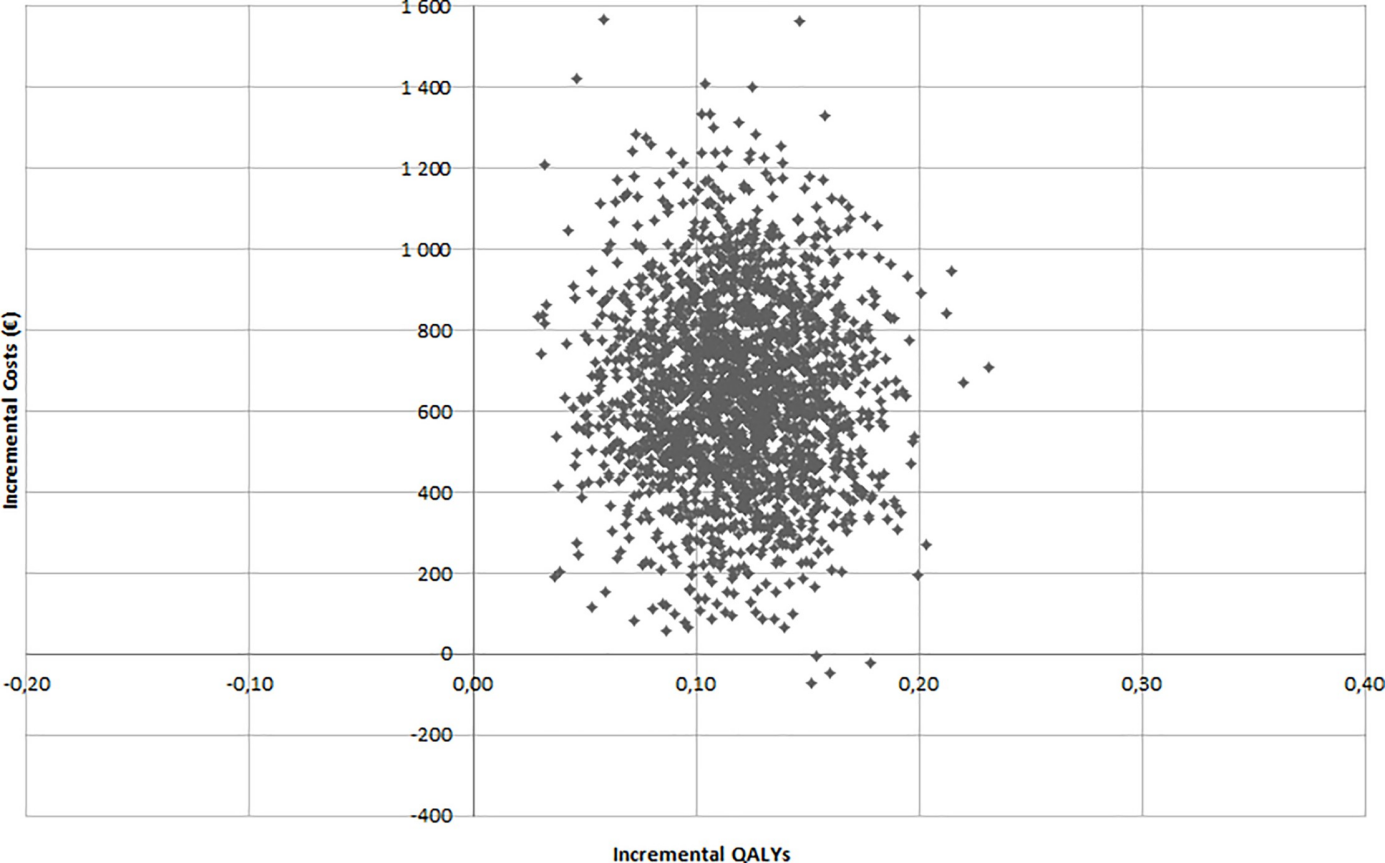
Probabilistic sensitivity analysis results (incremental cost-effectiveness plan). Abbreviation: QALY, quality-adjusted life-year.

**Fig 4:**
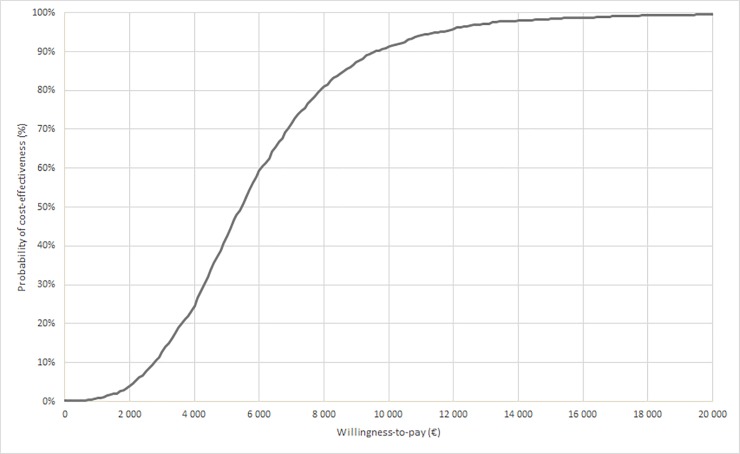
Cost-effectiveness acceptability curve. Abbreviation: QALY, quality-adjusted life-year.

Uncertainty around the cost-effectiveness results was demonstrated by plotting simulations from the PSA on a cost-effectiveness plane (Fig 3). All the probabilistic simulations suggest a QALY gain with rivaroxaban associated with an increase in cost for rivaroxaban (upper-right-hand quadrant) in 99.7% of the cases. The threshold corresponding to an 80% probability of rivaroxaban being cost-effective was estimated to €8,600 (Fig 4).

Scenario analyses were run to further test the robustness of the results. First, applying a utility decrement associated with rivaroxaban and VKA therapy reduced the incremental ratio to €5,495 per QALY gained. Second, considering treatment incident studies only in the meta-analysis reduced the incremental ratio to €5,471 per QALY gained. Thirdly, assuming no patient switch after discontinuation of initial treatment yielded to an incremental ratio of €5,507 per QALY gained while the opposite scenario assuming all patients switch, yielded to an incremental ratio of €6,228 per QALY gained. Finally, assuming all hazard ratios were taken from ROCKET-AF instead of RWE meta-analysis, increased the ICER up to €10,247.

## Discussion

Although there is no official willingness-to pay threshold in France, the above-mentioned results suggest that in the French healthcare setting, rivaroxaban could be considered as a cost-effective alternative to VKA for the prevention of stroke in AF patients, as a result of the clinical benefit shown in real-world setting. The model demonstrated that the benefits in terms of life expectancy and QALY gain were maintained over a lifetime horizon of 30 years, confirming the advantages of the strategy with rivaroxaban over VKA. Sensitivity analyses showed that uncertainty and variability in model parameters had a limited impact on results with all ICERs remaining below €11,000/QALY, suggesting a robustness of the conclusion.

To our knowledge, this analysis is the first model-based cost-effectiveness evaluation of a NOAC considering multiple RWE sources to document effectiveness (VKA rates and comparative effectiveness). A previous systematic literature review identified 18 cost-effectiveness models comparing rivaroxaban and other NOACs with VKAs for stroke prevention in patients with AF; all models derived safety and efficacy data from RCTs [37]. While the heterogeneous nature of clinical trials and modelling methods made it challenging to identify the most cost-effective agent amongst the NOACs, this review concluded that NOACs were frequently cost-effective compared to VKAs [37]. In France, two previous studies identified NOACs as cost-effective with incremental cost-effectiveness ratios below €20,000 per QALY gained compared to VKA [38] [25]. In the latter, uncertainty around RCT data used for NOACs was specifically highlighted, suggesting the need to validate the results in a French real-life context. Other published models considered RWE to document effectiveness [39–42], but none considered a meta-analysis with RWE only.

This model demonstrated a major improvement compared to the already published model. The model was also able to capture the different treatment regimens with the time on initial treatment considered through time-dependent rates, resulting in a complete discontinuation or a switch to another treatment. Additionally, RWE was considered as a key source for all model inputs, to fully reflect patients’ characteristics and disease progression seen in routine practice settings. This included population characteristics, clinical event rates, treatment effect, discontinuation, switch rates, utility, resource use, and unit costs for all events. This was considered to be the definition of a RWE cost-effectiveness model.

The use of RWE is associated to many challenges, such as dealing with the selection bias, missing data, lack of accuracy related to drug exposure and outcomes, errors during the record-keeping process [43], and also to potential benefits. For example, it would enable a shift from surrogate outcomes to clinical and long-term outcomes and constitute an important source of information in the specific case of rare harms. It is expected that methodologies used to analyse and synthesize RWE will continue to evolve. However, little guidance is available on the relative merits of using RWE in the modelling context [43–45].

The approach presented here is not without limitations. First, the model provides a simplification of what is expected in reality. Long-term outcomes believed to reflect the consequences of AF based on a number of assumptions to capture natural disease progression. Second, in the absence of France-specific utilities, this analysis considered values based on European data, assuming a reasonable similarity with French population. Of note, the deterministic sensitivity analysis did not report any significant impact of changes in utility values on the model’s conclusions. Third, the persistence rate from the VKA arm was taken from an US study [14] as no relevant French data were identified. However, the proportion of switch among the patients who discontinued their initial treatment was taken from a French study focussing on primary care [15]. Finally, multiple health states exist in the model with the possibility for patients to transition between all of them. This flexibility has a limitation as it allows patients to recover from major events by experiencing minor ones. As an example, a patient in post-major stroke could experience a minor stroke, and be associated to higher utility and lower costs. As this underestimates the burden of patients with VKA, it also underestimates the potential benefit associated with rivaroxaban.

## Conclusion

This RWE cost-effectiveness model in the context of stroke prevention in patients with AF demonstrated that rivaroxaban is likely to be cost-effective vs VKA in France in a real-world setting.
